# Analysis of the length polymorphisms in sequence-tagged-site sY1291 on Y chromosome in Vietnamese men of infertile couples

**DOI:** 10.1038/s41598-019-45649-3

**Published:** 2019-08-20

**Authors:** Nguyen Thi Tai Cao, Kien Trung Nguyen, Nhuan Thi Vu, Vieng Chung Nguyen, Thiet Minh Trinh, Ngoc Thi Bich Nguyen, Lien Thi Bich Trinh, Tien Thi Thuy Lam, Binh Luong Cao, Tra Ngoc Dang

**Affiliations:** 1 Department of Biology – Genetics, Faculty of Basic Science, Can Tho University of Medicine and Pharmacy, Can Tho city, Vietnam; 2 Department of Physiology, Faculty of Medicine, Can Tho University of Medicine and Pharmacy, Can Tho city, Vietnam; 3 Faculty of Endocrinology, Can Tho Obstetrics and Gynecology Hospital, Can Tho city, Vietnam; 4 Department of Foreign language, Faculty of Basic Science, Can Tho University of Medicine and Pharmacy, Can Tho city, Vietnam; 5 Department of Biology – Technology, Quoc Hoc Quy Nhon Highschool, Quy Nhon City, Vietnam

**Keywords:** DNA sequencing, Genetic markers

## Abstract

This study aims to analyze the length polymorphisms in sequence-tagged-site (STS) sY1291 of the Y chromosome in Vietnamese men of infertile couples. All 322 DNA samples were amplified with the sY1291 primer by the quantitative fluorescent polymerase chain reaction (QF-PCR) assay. DNA sequencing technique was employed to evaluate the accuracy of QF-PCR results. The study showed 273 out of 322 DNA samples had the presence of STS sY1291, accounted for 84.78%. The QF-PCR results showed that there were various lengths in STS sY1291: 507 bp, 512 bp, 523 bp and 527 bp. The most prevalent length in STS sY1291 was 507 bp (87.5%), the others were 512 bp (4.8%), 523 bp (4.8%) and 527 bp (2.9%). We found that the observed length polymorphisms derived from differences in the number of mononucleotide Thymine (T) repeats in its structure. It stretched from 22 T to 39 T. DNA sequencing results identified that the number of mononucleotide T repeats causes these polymorphisms. However, the pair-wise alignment between the obtained and reference sequence was 77%. It can be seen that the length polymorphisms in STS sY1291 observed in QF-PCR results was accurate but it is still difficult to sequence fragments with mononucleotide repeats.

## Introduction

The Y chromosome has 60 Mb in length, being one of the smallest chromosomes in human^1^. There are two regions on Y chromosome: pseudo-autosomal and non-combining region (also known as the male-specific region). The male-specific region of Y chromosome (MSY) comprises long Y-specific repeats called amplicons.

The sY1291 primers were selected to amplify STS sY1291 starting from 23,358,923 bp to 23,359,449 bp on Y chromosome (https://www.ncbi.nlm.nih.gov/nuccore/G72340). There are mononucleotide T repeats in this region. Its Gen Bank sequence (accession number G72340) has a stretch of 39 T.

While setting up a 14-plex-QF-PCR protocol to detect common genetics causes in men of infertile couples, we observed the length polymorphisms in STS sY1291. Previous studies have mentioned the length polymorphisms in STS sY1291, changing from 517 bp to 580 bp^2,3^.

Gel electrophoresis is not suitable for separating DNA fragments with small length differences. The best tool to solve this problem is capillary electrophoresis. This means DNA fragments with length polymorphisms can be easily detected by QF-PCR technology. The QF-PCR assay uses fluorescent-labeled primers to amplify the DNA fragments followed by analysis of capillary electrophoresis. This method relies on the amplification of polymorphic short tandem repeats (STRs) specific to chromosomes 13, 18, 21 and sex chromosomes^4^.

The amplified sequence of sY1291 was a non-polymorphic sequence. Blast sY1291 primers on the NCBI (GRCh38.p7), they amplify not only a fragment of sY1291 but also many fragments in PCR products. That is the reason why DNA sequencing was applied to check the preciseness of QF-PCR results. Sequencing technology has been used to confirm the homology of obtained and reference sequences. The goal of this study is to identify whether the QF-PCR results for the length polymorphism in STS sY1291 in men of infertile couples are correct or not by sequencing analysis.

## Results

### Patients’ clinical data

Among 322 men of infertile couples involved in the study (average age of 31.62 ± 5.53), 93 were azoospermia (28.8%) and 229 patients (71.2%) were severe oligospermia (a sperm count of less than five million per milliliter of semen). Follicle-stimulating hormone (FSH), luteinizing hormone (LH) and testosterone levels were divided into 2 groups: normal and abnormal. The increased and decreased hormone concentrations were put into the abnormal group. An overview of some characteristics of the men of infertile couples was presented in Table 1. STS sY1291 fragments were divided into two groups as negative and positive. Data were analyzed using Pearson chi-square for the correlation between hormone parameters (FSH, LH, and testosterone), sperm concentration, and STS sY1291 fragments. Azoospermic males and abnormal FSH concentration had significantly greater odds of negative STS sY1291 fragment (OR = 2.56, 95% CI = 1.37–4.78, and OR = 2.13, 95% CI = 1.15–3.96, respectively), compared with those with positive STS sY1291 fragments (Table 1).

**Table 1:** Overview of some characteristics of the men of infertile couples.

Patients’ clinical data	Negative STS sY1291 fragment (n = 49)	Positive STS sY1291fragment (bp)				OR (CI95%)	p value, χ^2^
		507 bp (n = 239)	512 bp (n = 13)	523 bp (n = 13)	527 bp (n = 8)		
**Sperm concentration**							
Azoospermia	23	62	5	2	1	2.56 (1.37–4.78)	0.004 9.17
Severe oligospermia	26	177	8	11	7		
**FSH level**							
Abnormal	23	65	8	2	5	2.13 (1.15–3.96)	0.02 5.94
Normal	26	174	5	11	3		
**LH level**							
Abnormal	4	18	0	1	0	1.18 (0.38–3.65)	0.76 0.09
Normal	45	221	13	12	8		
**Testosteron level**							
Abnormal	12	36	2	3	0	1.83 (0.88–3.81)	0.14 2.71
Normal	37	203	11	10	8		

OR: Odds Ratios; CI 95%: 95% Confidence Intervals; p < 0.05, significant.

### QF-PCR results

**Figure 1:**
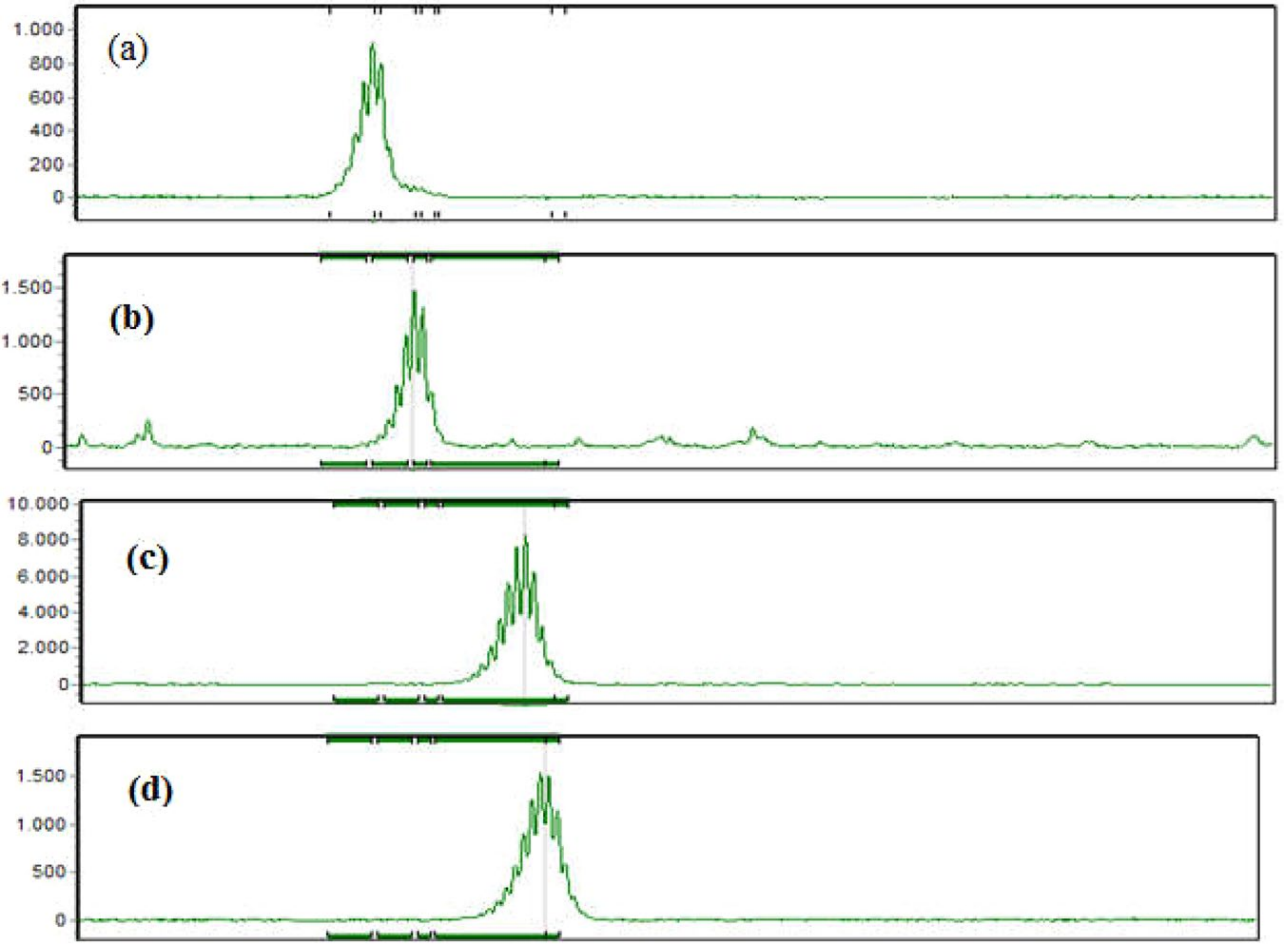
The length polymorphisms in STS sY1291 detected by the QF-PCR assay were 507 bp (**a**), 512 bp (**b**), 523 bp (**c**) and 527 bp (**d**).

Among 322 men of infertile couples, 273 cases (84.78%) had STS sY1291 fragment on their Y chromosome. The length polymorphisms in STS sY1291 in Vietnamese patients are illustrated in Fig. 1(a–d) with 507 bp, 512 bp, 523 bp, and 527 bp.

The QF-PCR results presented that there were various lengths in STS sY1291.They were 507 bp, 512 bp, 523 bp, and 527 bp. As summarized in Table 2, we presented the frequency of the length polymorphisms in STS sY1291. The most prevalent length in STS sY1291 was 507 bp (87.5%), the others were 512 bp (4.8%), 523 bp (4.8%), and 527 bp (2.9%).

**Table 2:** Frequency of length polymorphisms in STS sY1291 detected by QF-PCR assay.

Marker name	Allele size range in bp (a reference size from NCBI, GRCh38.p7)	Allele size range in bp (the obtained sizes)	Number of cases n = 273 (%)
sY1291	527	507	239 (87.5)
		512	13 (4.8)
		523	13 (4.8)
		527	8 (2.9)

### Gel electrophoresis

**Figure 2:**
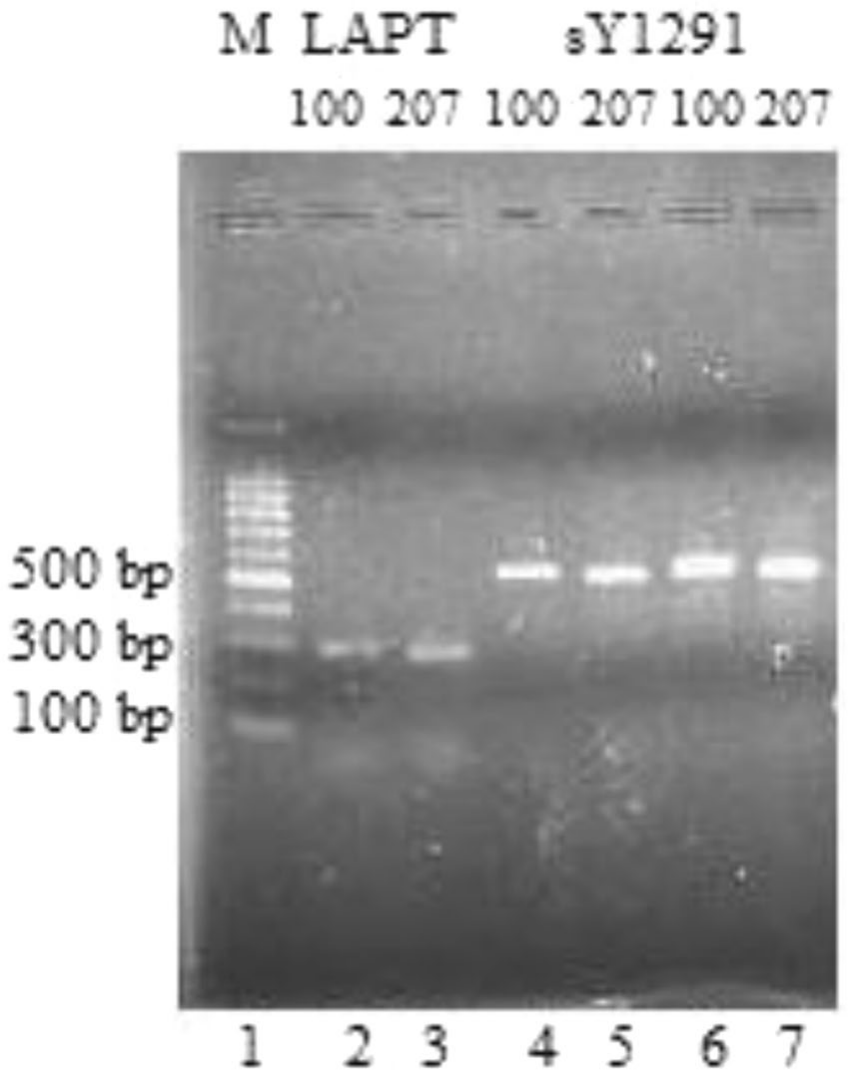
A representative of the PCR products on the agarose gel, lane 1- standard marker (M stands for marker), lane 2, 3- DNA of patients amplifying with the LAPT primers and lane 4–7- DNA of patients amplifying with sY1291 primers.

Samples amplified with sY1291 primers having 507 bp bands (sample 207 in lane 5, 7) and 527 bp bands (sample 100 in lane 4,6) were chosen to sequence. Gel electrophoresis was conducted to check the PCR products (Fig. 2). Besides, in Fig. 2, the samples amplified from LAPT primers presented approximately 300 bp bands in lane 2 and 3. According to the reference size on the NCBI (GRCh38.p7), the LAPT primer was designed to amplify a 288-bp-PCR-fragment that contains mononucleotide T repeats. The amplified fragments had length polymorphisms, so PCR products had different sizes compared to the reference size on the NCBI database.

**Figure 3:**
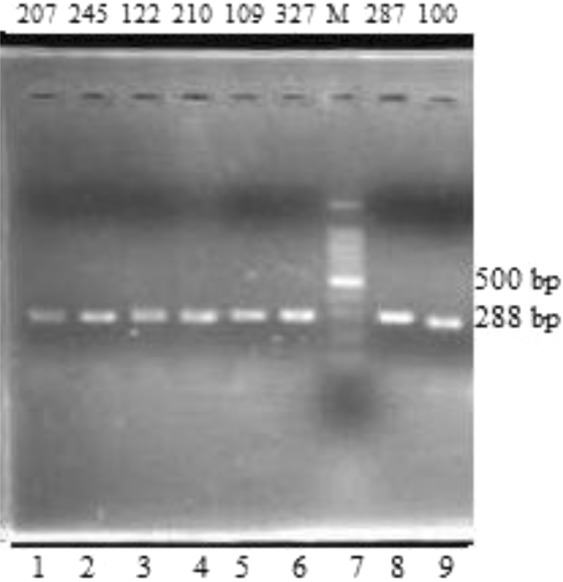
PCR products of LAPT fragments in all lanes (except a standard marker in lane 7).

The designed LAPT forward primer was 5′CAGAACTGCCAGGTCTGTGTCTTAT3′ and the reverse primer was 5′ACCATCCCGGCTAAAAACGGTG3′. As the data showed on the NCBI reference sequence, mononucleotide T repeats are in its structure. Therefore, the actual sizes of PCR products among samples are dependent on the times of mononucleotide T repeats. The lengths of LAPT fragments are presented in Fig. 3. However, we do not know exactly the PCR product lengths amplified from LAPT primers because it is not easy to observe the fragments with small differences on gel electrophoresis.

### Sequencing analysis

**Figure 4:**
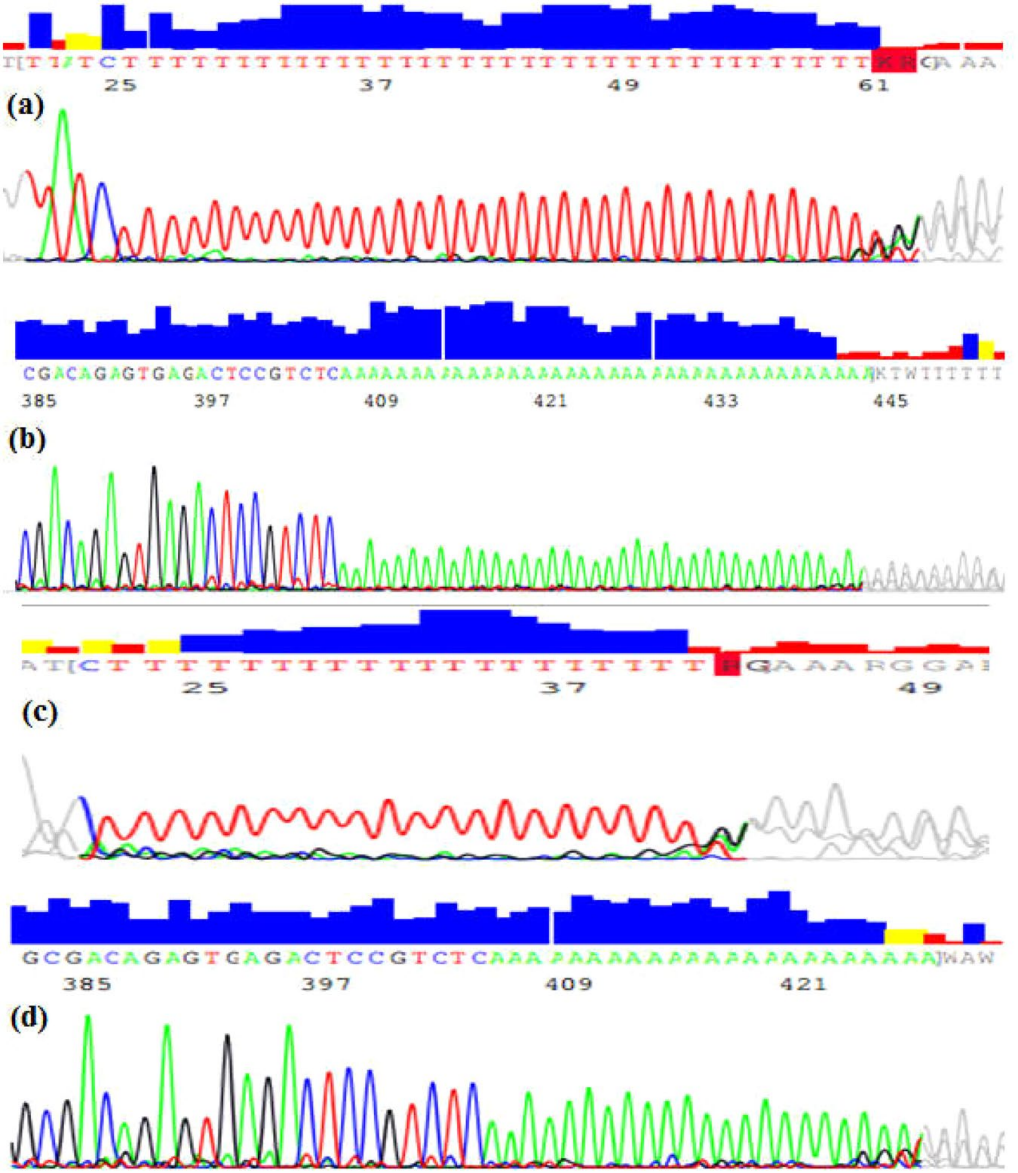
The sequencing results of STS sY1291 by Sequencing software 6 with a 527-bp of forward fragment (**a**), a 527-bp of reverse fragment (**b**), a 507-bp-sY1291 forward fragment (**c**), a 507-bp-sY1291 reverse fragment (**d**).

To confirm of the different sizes seen in STS sY1291 in QF-PCR results, sequencing analysis was performed. Samples with 507 bp band and 527 bp band were sequenced on both forward and reverse STS sY1291. The sequencing results are shown in Fig. 4(a–d).

**Figure 5:**
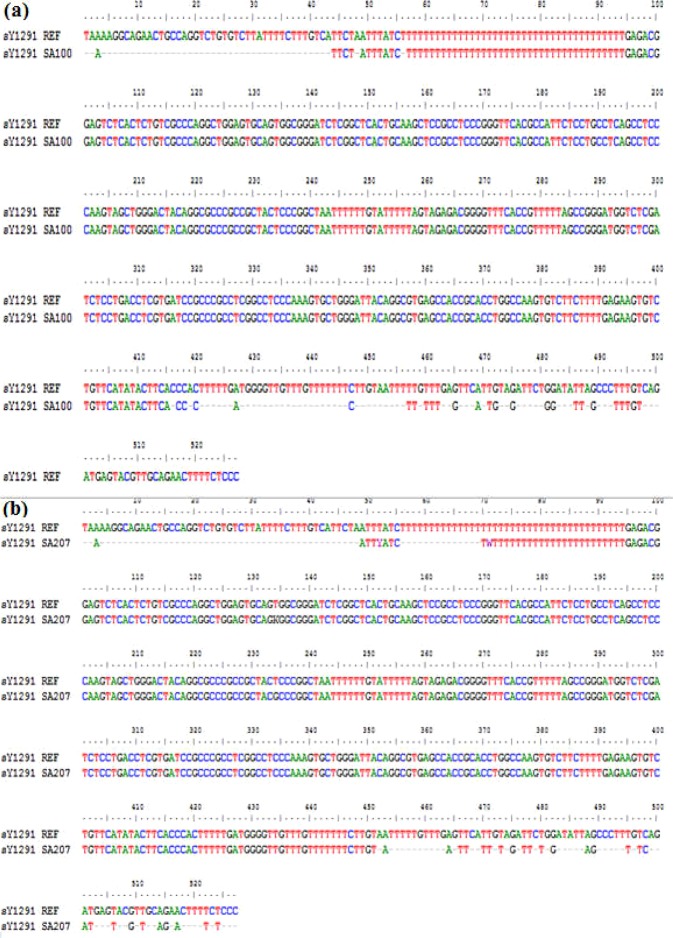
Identities between a reference sequence (REF) and a sample sequence (SA100) with a 527-bp-PCR product (**a**), and a sample sequence (SA207) with a 507-bp-PCR product (**b**).

The sequencing results on both forward and reverse sequences showed slippage problems after T tracts. Hence, by using Bioedit software V7.2.5, the pair-wise alignment was 77% between the obtained and reference sequences (Fig. 5(a,b)).

**Figure 6:**
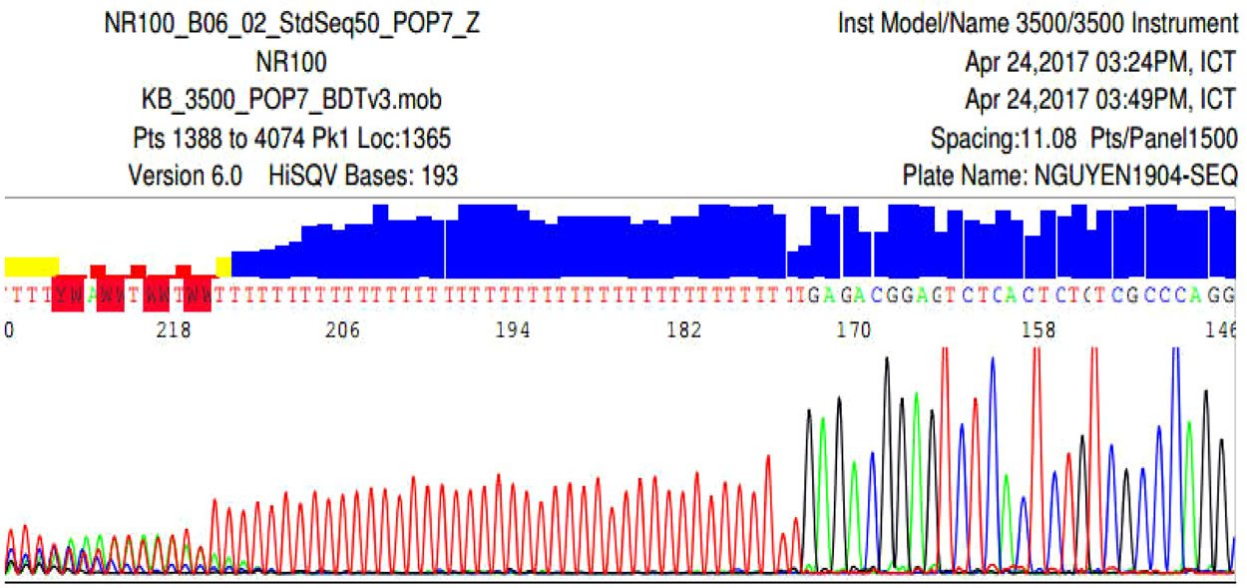
The sequencing result of the reverse fragment with mononucleotide T repeats.

Similarly, the sequencing results of fragments amplified by LAPT primers had the same problem. A slippage problem after T tract was observed as shown in Fig. 6.

As summarized in Table 3, the times of mononucleotide T repeats were observed in obtained sequences amplified from LAPT and sY1291 primers. The results showed that the length polymorphisms had 25 to 39 mononucleotide T repeats.

**Table 3:** Comparison of the number of mononucleotide T repeats amplified from LAPT and sY1291 primers.

The length polymorphisms of PCR fragments	Mononucleotide T repeats (NCBI, GRCh38.p7)	Mononucleotide T repeats Observed in LAPT and STS sY1291	
		LAPT	sY1291
507 bp	/	25	22
512 bp	/	25	/
523 bp	/	36	/
527 bp	39	39	37

## Discussion

There were 322 men of infertile couples with a sperm count of less than five million per milliliter of semen. Among them, the lowest age was 18 years old, the highest was 49 years old and the average age was 31.62 ± 5.53, which is consistent with many studies of average age from 30 to 35 years^5,6^. In contrast, other studies reported an average age of over 35 years^6–12^ (Table 4).

**Table 4:** Comparation age of azoospermic and oligospermic patients among the studies.

Authors	Population	Sperm count (million/ml)	n	Age	Mean
Majumder *et al*. (2013)	Syrian	<5	162	17–45	32±7.9
Amouri *et al*. (2014)	Tunisian	≤5	476		37.94 ± 3.00
Gallego *et al*. (2014)	Spanish	0–0.25	11		36.8
Lei *et al*. (2015)	Chinese	0	471	18–63	31.01
Evangelini *et al*. (2016)	Egyptian	≤1	114	15–53	36
Suganya *et al*. (2016)	Indian	≤5	228	20–53	35.4 ± 6
Saad *et al*. (2017)	Tunisian	≤15	163	22–62	37
This study	Vietnamese	≤5	322	18–49	31.62 ± 5.53

The study findings showed that azoospermic males and abnormal FSH levels had significantly greater odds of negative STS sY1291 fragment (OR = 2.56, 95% CI = 1.37–4.78, and OR = 2.13, 95% CI = 1.15–3.96, respectively), with p < 0.05. However, there has not been any research mentioning these correlations yet. From these results, further research should be done to have a clear conclusion.

Our study found that STS sY1291 was polymorphic in size due to various lengths of a poly-T stretch (507–527 bp) in Vietnamese patients. This finding was in concordance with two previously reported instances in which Lin *et al*. (2006) reported STS sY1291 to be polymorphic (565–580 bp) in Han Chinese and Evguenia (2016) showed different sizes of STS sY1291 between haplogroups Q1a3a1 (517 bp) and R1b1a2 (538 bp) in a Native American population^2,3^. It is important to highlight that our studies used the same primers sequences to amplify this STS, but the lengths of STS sY1291 on Y chromosome differed from the previous studies. So far, STS sY1291 length polymorphism has been reported in only two studies. The result matches the other studies showing that the differences rely on countries, races, and ethnicities.

**Figure 7:**
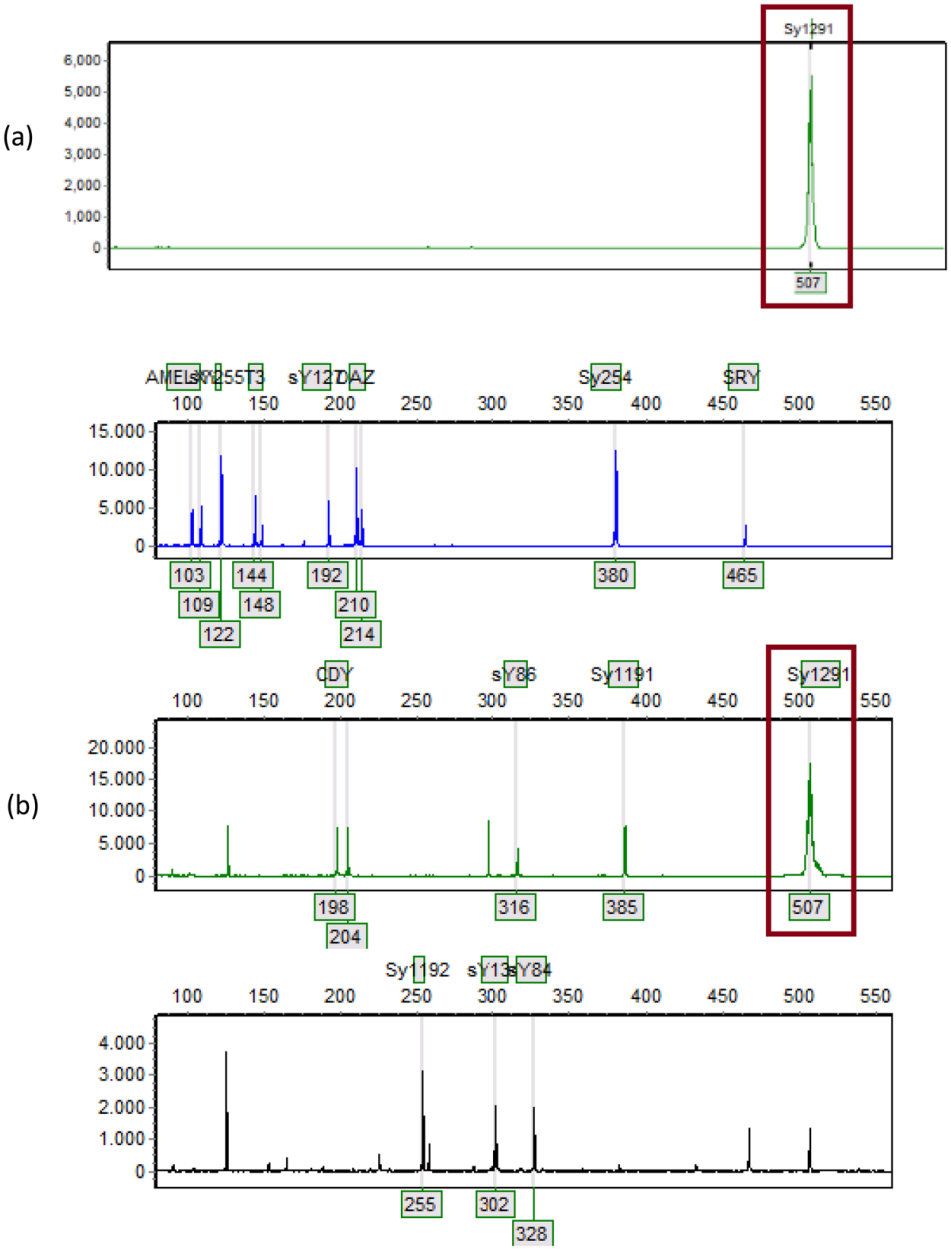
PCR product with individual sY1291 marker (**a**), and with 14-plex PCR (**b**) are of the same length, recognized at red rectangles of 507 bp.

We observed four lengths in STS sY1291 in Vietnamese patients. The frequency of length polymorphisms in STS sY1291 detected by QF-PCR assay was 507 bp (87.5%), 512 bp (4.8%), 523 bp (4.8%), and 527 bp (2.9%). As mentioned in the introduction, the QF-PCR assay has 14 markers (sY84, sY86, sY127, sY134, sY254, sY255, sY1191, sY1192, sY1291, SRY, DAZ, CDY, T3 (TAF9B3/TAF9BX), AMELXY), the capillary electrophoresis presented that the results of running individual sY1291 PCR product matches those of pooling the 14 PCR products (Fig. 7).

We have not found any studies mentioned the length polymorphisms in Vietnamese STS sY1291 yet. Briefly, 507-bp-STS sY1291 is the most popular among Vietnamese severe oligospermia and azoospermia. While our findings show the polymorphism among the men of infertile couples, no data are presented regarding the findings in the normal populations yet. Therefore, this is a limitation of our study. Further analysis should be conducted on Vietnamese healthy men to compare STS sY1291 length polymorphisms between groups and to determine whether these polymorphisms are related to infertility or not.

To confirm our QF-PCR results, the amplification products for STS sY1291 had been sequenced. As shown in Fig. 4(a–d), the results presented a mixed signal downstream of the poly-T stretch. This phenomenon is called DNA sequence slippage problems. Up to now, the cause of DNA sequence slippage problems is the sequencing DNA polymerase skips over one or more nucleotides. In theory, STS sY1291 has 39 mononucleotide T repeats; therefore, DNA polymerase will slip on a long repeat region. That is why the lengths of DNA sequencing products were changed. Mononucleotide repeats makeup blocks of identical base pairs (A/T or C/G, referred to as T-tracts) and display distinct features sequence polymorphism within the general population has been linked to phenotypic diversity^4,13,14^. Both historic and recent investigations concur with the conclusion that a major source of mononucleotide repeats is the occurrence of slippage during semi-conservative DNA replication, which gives rise to the addition or deletion of repeat units^13,14^.

STS sY1291 has long mononucleotide T repeats in its structure. As shown in the sequencing results, the unclear peaks appeared after long mononucleotide repeats. Hence, the pair-wise alignment between the obtained and reference sequences was 77%. The current results, together with previous findings, allow us to conclude that it is difficult to have a higher homology in this problem.

The results showed that the observed polymorphisms stemmed from differences in the number of mononucleotide T repeats in the stretch that ranged from 22 T to 39 T. Evguenia (2016) reported that STS sY1291 had 16 T in haplogroup Q1a3a1 (517 bp) and 39 T in haplogroup R1b1a2 (538 bp). Comparing to Evguenia’s study, our research was presented 22 T (507 bp) and 37 T (527 bp) with the same primers sequences^2^. Our designed primers to figure out mononucleotide T repeats among samples had shown 25 T (507 bp and 512 bp), 36 T (523 bp), and 39 T (527 bp). In summary, it is not clear to count the mononucleotide T repeats on STS sY1291 although PCR products had the differences. More studies should be done to improve this long mononucleotide problem in STS sY1291.

## Methods

### Patients

The study was performed between November 2014 and March 2016 in Can Tho Obstetrics and Gynecology Hospital. A total of 322 Vietnamese men of infertile couples with azoospermia (n = 93) and severe oligospermia (a sperm count of less than five million per milliliter of semen, n = 229), aged between 18 and 49 (the mean ± SD 31.62 ± 5.53 years old), were included in the study. Semen analysis was performed according to the World Health Organization guidelines^15^.

A detailed medical history was recorded for each patient. The patients were also interviewed regarding their medical history, family background, and reproductive problems. Clinical data, semen, hormone analyses and blood samples were obtained from the Department of Infertility and Department of Medical Genetics, Can Tho Obstetrics and Gynecology Hospital.

Inclusion criteria were men of infertile couples (≥18 to 49 years old), capable of giving informed consent, having semen analysis with a sperm count of less than five million per milliliter of semen.

Exclusion criteria were a history of drug consumption, fever in the previous 6 months, inflammation of seminal vesicle, varicocele, systemic disease, previous cryptorchidism or orchitis, hypogonadotropic hypogonadism, treatment with chemotherapeutic agents or radiotherapy, testicular tumors^16^.

### DNA extraction

Extraction of 322 DNA was done with GeneAll kit (Korea), strictly following the manufacturer’s instructions.

### Hormone analyses

Serum was analyzed for FSH, LH, and testosterone using an automated chemiluminescence immunoassay (Cobas 6000 e 601; Roche Diagnostics), with the detectable ranges for FSH being 2–10 mIU/ml FSH.

### Quantitative fluorescent polymerase chain reaction assay

The primer pair to amplify DNA fragments containing STS sY1291 was described below. PCR amplification of DNA fragments was performed with forward (5′-TAAAAGGCAGAACTGCCAGG-3′) and reverse (5′-GGGAGAAAAGTTCTGCAACGT-3′) primers from ABI, USA. Forward primer was labeled with VIC fluorescent dye, which allowed the determination of the length of STS sY1291 on an Applied Biosystems Genetic Analyzer 3500 (USA) with a GeneScan™ 600 LIZ™ dye Size Standard v2.0 (Applied Biosystems, USA). The QF-PCR products were separated using POP-7 polymer and data collection software V1.0. Peak sizing was done by GeneMarkerV2.6.3 software.

All reactions were accomplished with 5 ml multiplex PCR 5X master mix (New England Biolabs, Inc.), 10 ng genomic DNA, 10pmol each of the primers, in a final volume of 25 ml. QF-PCR assay was performed in a PCR Mastercycle Pro S (Eppendorf) with the following cycling conditions: an initial denaturation step at 94 °C for 2 minutes, followed by 30 cycles of 30 seconds denaturation at 94 °C, 1 minute annealing at 58 °C and 1.5 minutes elongation at 68 °C; and final elongation at 68 °C for 10 minutes.

### DNA sequencing

The STS sY1291 was amplified by using the same reverse primer (5′GGGAGAAAAGTTC TGCAACGT-3′) in QF-PCR assay and the forward primer from Macrogen, Korea without labeled fluorescent dye.

We designed a forward and a reverse primer (named LAPT) that lies in the a 527-bp-STS-sY1291 region. The Y chromosome reference sequence from the NCBI database was searched for the STS sY1291 (http://www.ncbi.nlm.nih.gov/). Optimal LAPT primer was designed using Primers 3 software (http://frodo.-wi.mit.edu/primers31°. The predicted amplification product was checked using UCSC Genome Browser online software (http://genome.ucsc.edu/cgi-bin/hgGateway) and primer amplifying a 288-bp-amplicon was obtained. In order to obtain broader divergence between melting peaks, the amplicons melting temperature (Tm) was predicted using the Tm Calculator (http://tmcalculator.neb.com/#!/main). A designed LAPT forward primer (5′CAGAACTGCCAGGTCTGTGTCTTAT3′) and reverse primer (5′ACCATCCCGGCTAAAAACGGTG 3′) were produced from Macrogen, Korea.

Similarly, LAPT primer was conducted in the same condition with sY11291 primer, except the annealing at 60 °C. The PCR products were submitted on 1.5% agarose gel electrophoresis to size of products stained with safeview. They were then viewed under UV trans illumination. Photographs of the gel were taken using a gel documentation system.

ExoSAP-IT^®^PCR Product Cleanup (Affymetrix, Inc., USA) was employed to confirm the specifility of all PCR products before sequencing with both the forward and reverse primers using the BigDye^®^ Terminator v3.1 Cycle Sequencing (Life Technologies, USA), and sequencing purification using BigDyeXTerminator^®^ Purification Kit, according to the supplier’s protocol. Capillary electrophoresis of the sequences was carried out in an ABI3500 Genetic Analyzer (Life Technologies, USA) using a 36 cm array and POP7 polymer. Capillary electrophoresis conditions were 55 °C, 15 kV/180 sec (pre-run), 1.6 kV/8 sec (injection) and 15 kV/1700sec (run). Sequencing analysis was carried out with SecScape and Sequencher software. Mononucleotide T repeats polymorphisms among samples were determined by sequencing analysis software 6 and Bioedit software V7.2.5.

Samples of STS sY1291 507 bp and 527 bp fragments were chosen to amplify. LAPT primer was used to amplify samples having the sY1291 length polymorphisms including 507 bp, 512 bp, 523 bp, and 527 bp.

### Statistical analysis

Statistical analysis was done by SPSS for Windows, version 16. The data were presented as a range of number and percentage. The mononucleotide T repeats in sequencing results were calculated by counting. Data were analyzed using Pearson chi-square for the correlation between hormone parameters, sperm concentration, and 2 groups of STS sY1291 (negative and positive STS sY1291). A p value < 0.05 was considered statistically significant.

### Ethical approval and informed consent

This study was approved by the ethics committee of Can Tho Obstetrics and Gynecology hospital, Can Tho University and Can Tho Department of Science and Technology, Vietnam. All participants provided informed consent. All personal information was kept confidential.
